# An Inverted Repeat in the *ospC* Operator Is Required for Induction in *Borrelia burgdorferi*

**DOI:** 10.1371/journal.pone.0068799

**Published:** 2013-07-03

**Authors:** Dan Drecktrah, Laura S. Hall, Laura L. Hoon-Hanks, D. Scott Samuels

**Affiliations:** 1 Division of Biological Sciences, The University of Montana, Missoula, Montana, United States of America; 2 Center for Biomolecular Structure and Dynamics, The University of Montana, Missoula, Montana, United States of America; University of Kentucky College of Medicine, United States of America

## Abstract

*Borrelia burgdorferi*, the spirochete that causes Lyme disease, differentially regulates synthesis of the outer membrane lipoprotein OspC to infect its host. OspC is required to establish infection but then repressed in the mammal to avoid clearance by the adaptive immune response. Inverted repeats (IR) upstream of the promoter have been implicated as an operator to regulate *ospC* expression. We molecularly dissected the distal inverted repeat (dIR) of the *ospC* operator by site-directed mutagenesis at its endogenous location on the circular plasmid cp26. We found that disrupting the dIR but maintaining the proximal IR prevented induction of OspC synthesis by DNA supercoiling, temperature, and pH. Moreover, the base-pairing potential of the two halves of the dIR was more important than the nucleotide sequence in controlling OspC levels. These results describe a *cis*-acting element essential for the expression of the virulence factor OspC.

## Introduction

Lyme disease is caused by the spirochete *Borrelia burgdorferi*, which is transmitted via an *Ixodes* tick [1]–[3]. *B. burgdorferi* is maintained in an enzootic cycle between its tick vector and a vertebrate host reservoir [4]–[6]. Naïve larvae acquire *B. burgdorferi* when feeding on an infected animal and can then transmit the bacterium to uninfected hosts as nymphs during feeding, completing the cycle. *B. burgdorferi* encounters disparate, and hostile, environments as it transitions through the enzootic cycle; the spirochete has evolved a repertoire of strategies, which involve the regulation of a variety of genes, to respond and adapt to these changes during its lifecycle [6]–[8].

The differential syntheses of outer surface lipoproteins (Osp), which are the interface between *B. burgdorferi* and its hosts, is paramount to the ability to infect, survive and replicate in both the tick and the vertebrate [6]–[10]. These regulated genes encode wide-ranging functions; for example, VlsE mediates evasion from the vertebrate immune system [11]–[13], while OspA binds to a tick midgut protein and protects the spirochetes from the incoming blood meal [14]–[17]. One of the best-studied lipoproteins is OspC [18], which is required for transmission and the early stages of mammalian infection [19]–[22]. The *ospC* gene is carried on the conserved 26-kb circular plasmid cp26 [23], [24]. The precise function of OspC remains elusive, but the outer membrane lipoprotein appears to have several activities [25], including providing initial protection from the innate immune system [26] and facilitating dissemination [27]. Additionally, OspC has a ligand-binding domain essential for its function [28] and binds the tick salivary protein Salp15 [29] as well as mammalian plasminogen [30], [31], which may assist in transmission and dissemination, respectively. OspC is highly immunogenic, so its synthesis must be repressed for the spirochete to persist in the mammal [32]–[34]. *B. burgdorferi* that continue to produce OspC during infection of immunocompetent mice are cleared [34] and *ospC* expressed in *trans* from a shuttle vector is selected against during mammalian infection [35]. While *ospC* is present in all *B. burgdorferi* strains examined, the sequence is variable, with only strains carrying certain alleles capable of disseminating and establishing infection in humans [13], [36]–[39].

OspC synthesis is induced *in vitro* in response to increased temperature, which presumably mimics a signal that occurs during tick feeding [40]–[42]. Subsequent studies showed that numerous factors such as pH [43], [44], DNA supercoiling [42], oxygen [45], carbon dioxide [46], acetate [47], and transition metals [48] also control *ospC* expression. External signals are transmitted through a unique signaling pathway involving the sequential action of two alternative sigma factors, RpoN (σ^54^) and RpoS (σ^s^) [49], [50]. RpoN, in collaboration with the response regulator Rrp2 [51]–[54] and the transcription factor BosR [55]–[58], activates transcription of *rpoS*; RpoS, in turn, activates transcription of *ospC* [49] and other genes [54], [59], [60]. Another level of control of *ospC* expression is exerted during translation of *rpoS*. The small RNA DsrA_Bb_ [61], [62] and the RNA chaperone Hfq [63] control *rpoS* translation and subsequently *ospC* transcription. However, there is no evidence for post-transcriptional control of the *ospC* gene [42], [44]–[49], [52], [61], [64].

Previous studies identified an *ospC* operator consisting of two overlapping inverted repeats (IRs) 42 bp upstream of the major transcriptional start site [23], [65]–[67]. The operator is highly conserved in *B. burgdorferi* sensu stricto [67], but there is some controversy about its role in *ospC* gene regulation. Eggers et al. [64] showed that the operator is required for full *ospC* expression *in vitro*, while Yang et al. [68] found the operator is dispensable for induction and repression. In addition, mutants lacking the operator were unable to persist in immunocompetent mice and were also cleared from SCID mice injected with transferred anti-OspC antibodies, suggesting that the operator is required to repress *ospC* expression during infection [69]. One caveat to these previous studies is that *ospC* was expressed in *trans* in an *ospC* null background strain. In the current study, we molecularly dissect the *ospC* operator in its native locus on cp26 and show that the distal IR is an important *cis*-acting element controlling OspC expression.

## Materials and Methods

### Bacterial Strains and Culture Conditions

Low-passage *B. burgdorferi* strain 297 (BbAH130) [49] and all mutant strains were maintained in Barbour-Stoenner-Kelly II (BSK-II) liquid medium, pH 7.6, containing 6% rabbit serum [70]. In temperature shift experiments, cultures were passaged twice at 23°C, before inoculating cultures at 1×10^5^ cells ml^−1^ and growing at 23°C to late log phase (5 to 9×10^7^ cells ml^−1^) or inoculating cultures at 1×10^3^ cells ml^−1^ and growing at 34°C to late log phase. For temperature downshift experiments, cultures were grown to log phase at 34°C before inoculating cultures at 1×10^5^ cells ml^−1^ and growing at 23°C to late log phase. In experiments examining the effect of pH, cultures at 34°C in BSK II at pH 8.0 were passaged into BSK II at pH 7.0. To determine the effect of DNA supercoiling, cultures were grown at 23°C (inoculated at 1×10^5^ cells ml^−1^) in the presence of 10 ng ml^−1^ coumermycin A_1_ (50 mg ml^−1^ stock in DMSO) or DMSO (solvent control) until late log phase [42]. Cell density was determined using a Petroff-Hausser counting chamber [71].

### Construction of *ospC* Promoter Mutants

Mutations in the *ospC* operator were generated by allelic exchange on cp26 [72]. Constructs containing the operator mutants were made by overlap extension PCR [73]. The 5′ portion of the upstream construct was amplified by PCR of genomic DNA using KOD polymerase (Novagen) with the primers ospC U866F and ospCp mutHup(297)R or ospCp mutDH(297)R2 (Table 1). The 3′ portion of the upstream construct, which also includes the *ospC* gene, was amplified by PCR using the primers ospC D697R+AatII+AgeI and ospCp mutHup(297)F (Table 1). PCR products were separated and purified in a 1% agarose gel. Paired 5′ and 3′ portions of the upstream construct were then combined and extended for six cycles in a thermal cycler. Next, the primers ospC U866F and ospC D697R+AatII+AgeI (Table 1) were added and the combined upstream construct was amplified by PCR. To generate the construct for the wild-type control strain with the antibiotic resistance cassette, PCR was done using primers ospC U866F and ospC D697R+AatII+AgeI with genomic DNA as a template. PCR products were separated in a 1% agarose gel, gel purified, polyadenylated, and cloned into pCR®2.1-TOPO. PCR of genomic DNA with the primers ospC D673F+AatII and ospC D1572R+AgeI (Table 1) was used to amplify the downstream construct, which was cloned into pCR®2.1-TOPO as described above. The accuracy of all DNA constructs was confirmed by sequencing. The downstream sequence was inserted into the upstream *ospC* operator mutation constructs using the synthetic AatII and AgeI restriction sites. Lastly, the kanamycin resistance cassette *flgBp*-*aphI* [74] was inserted downstream of the *ospC* gene into the engineered AatII site. The orientation of *flgBp*-*aphI* was determined by PCR using the primers kanR 488R and ospC D1572R+AgeI (primers a and b, respectively). The plasmid was linearized by digestion with AhdI and electroporated into competent *B. burgdorfer*i [61], [71], [72]. Transformants were cloned in liquid BSK-II by diluting the electroporated cells to less than one cell per well of a 96-well plate in medium containing kanamycin (200 µg ml^−1^) at 34°C and a 1.5% CO_2_ atmosphere [16]. Total genomic DNA was isolated from positive colonies and sequenced by the Murdock DNA Sequencing Facility at The University of Montana using the primer ospC U291F to confirm the site-directed *ospC* operator mutations.

**Table 1:** Oligonucleotides used in this study^a^.

Name	Sequence (5′-3′)
ospC U866F	AGCTTAATTTTTTCCACAATGG
ospC D697R+AatII+AgeI	ACCGGT AATGACGTCTGACTTATATTGACTTTATTTTTCCAG
ospC D673F+AatII	GACGTC GGAAAAATAAAGTCAATATAAGTCAAG
ospC D1572R+AgeI	ACCGGT AATGGAAAAATTCCTAATGTCG
ospC U291F	ATTAGTTGGCTATATTGGG
kanR 488R	TCACTCGCATCAACCAAACC
ospCp mutHup(297)F	TAAGACAATATTGAAAAAATTCTTCAAT
ospCp mutHup(297)R	ATTGAAGAATTTTTTCAATATTGTCTTA
ospCp mutDH(297)R2	TTTCAATTTTTTATTTTTTCAAATATTTGAAAAAATTCTTCAATATTT
flaB 423F	TTCTCAAAATGTAAGAACAGCTGAAGA
flaB 542R	TGGTTTGTCCAACATGAACTC
flaB probe	6-FAM-TCACTTTCAGGGTCTCAAGCGTCTTGGAC-TAMRA
ospC F	CATGGGCAACTTGGAATTGA
ospC R	TTGCCAAGTTTTCTACTGCTTTAAATAG
ospC probe	6-FAM-TAAAGATAAGGGCGCTGCAGAGC-TAMRA

a Synthetic restriction sites are underlined.

### RNA Isolation and qRT-PCR Analysis

RNA was isolated from 40-ml cultures of *B. burgdorferi* grown at 23°C containing 10 ng ml^−1^ coumermycin A_1_ or DMSO as a solvent control using TRIzol (Invitrogen) as previously described [61], [75]. Samples were treated with Turbo DNase (Ambion) to remove contaminating DNA. Samples were screened by PCR to ensure that contaminating DNA had been removed using the primers flaB 423F and flaB 542R. cDNA was synthesized using 1 µg RNA with SuperScript^®^ III for qRT-PCR (Invitrogen). *flaB* and *ospC* primers and probes were designed using Primer Express^®^ version 3.0 (Applied Biosystems). TaqMan absolute qRT-PCR was performed in 96-well plates using an Applied Biosystems 7300 Real-Time PCR System and standard curves for *flaB* and *ospC* were generated using a portion of the *flaB* ORF (nucleotides 278–551 of the ORF) cloned into pCR®2.1-TOPO and *B. burgdorferi* strain 297 (BbAH130) genomic DNA, respectively. Values represent the mean (±SE) from three independent experiments.

### SDS-PAGE and Immunoblotting

*B. burgdorferi* cultures were grown to late log phase at 23°C or 34°C and total cell lysates collected as previously described [61], [72]. Equal amounts of protein were separated on pre-cast Novex 4–20% Tris-Glycine polyacrylamide gels (Invitrogen). Proteins were transferred by electroblotting to PVDF Immobilon™ membranes (Millipore) and membranes blocked in Blocking Buffer (138 mM NaCl, 2.7 mM KCl, 8.1 mM Na_2_HPO_4_, 1.5 mM KH_2_PO_4_, 0.05% Tween 20, 4% dry milk, and 1% goat serum) overnight at 4°C. Membranes were incubated with rabbit anti-OspC antibodies (1∶1000) [68], [76] or anti-FlaB antibodies (1∶1000) (a kind gift from Tom Schwan) in Blocking Buffer for 1 h at room temperature. Rabbit antibodies were detected by incubating membranes with goat anti-rabbit HRP-linked antibodies (Bio-Rad Laboratories) (1∶20,000) in Blocking Buffer for 1 h at room temperature. HRP-linked antibodies were visualized by chemiluminescence using Amersham™ ECL Plus (GE Healthcare) and images were collected using an LAS-3000 Intelligent Dark Box (Fujifilm Medical Systems USA). Images were processed using ImageJ (US National Institutes of Health and available at http://rsbweb.nih.gov/ij/) and Pixelmator (Pixelmator Team, Ltd).

## Results

**Figure 1:**
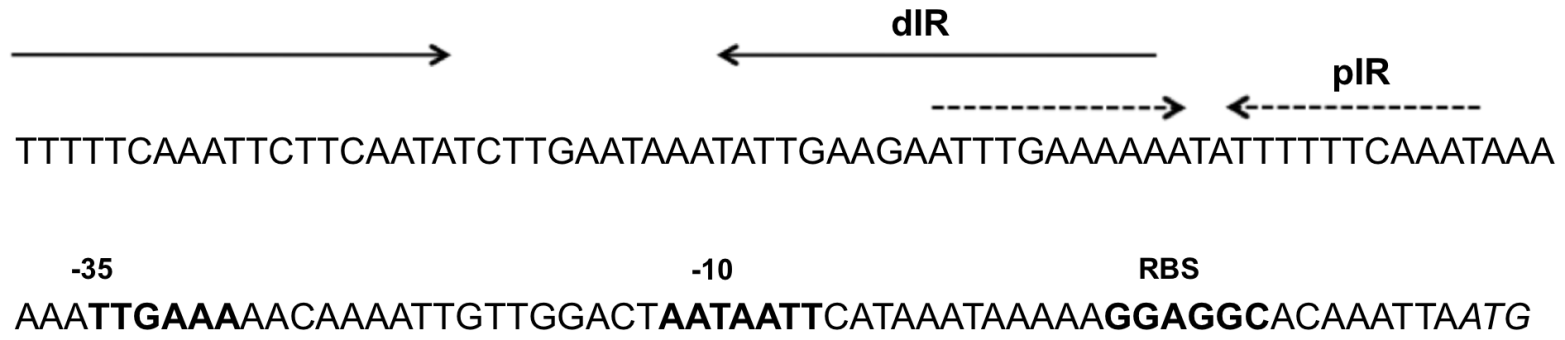
The sequence of the *B. burgdorferi* strain 297 *ospC* operator. Two overlapping inverted repeats (IR): proximal (pIR, dashed arrows) and distal (dIR, solid arrows) of the *ospC* operator. The promoter (−10 and −35) and ribosome-binding site (RBS) are in bold and the translational start site is in italics.

**Figure 2:**
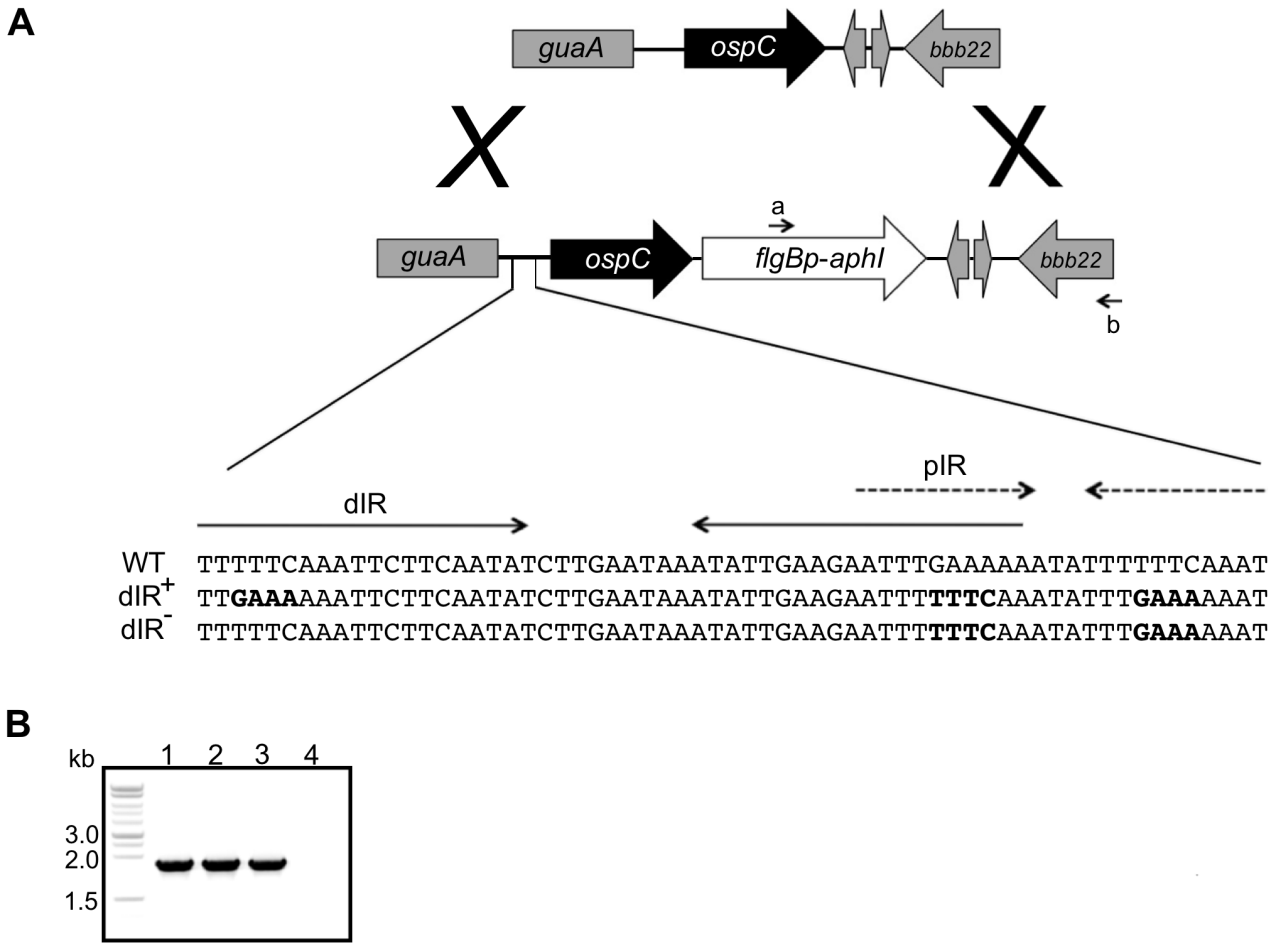
The *ospC* operator and mutagenesis strategy. (A) *ospC* operator mutations are linked to the kanamycin resistance cassette (*flgBp-aphI*). The sequence upstream of the *ospC* gene in *B. burgdorferi* strain 297 is shown (WT) with the dIR (solid arrows) and the overlapping pIR (dashed arrows). The nucleotides that have been changed are marked in bold. The strain nomenclature is as follows: dIR^+^ has the nucleotide sequence changed but the complementarity of the inverted repeats maintained, and dIR^−^ has the distal inverted repeat disrupted but the complementarity of the proximal IR intact. (B) PCR of genomic DNA from *ospC* operator mutants (lane 1, WT with *flgBp*-*aphI* cassette; lane 2, dIR^+^; lane 3, dIR^−^; and lane 4, no template control) using primers primers kanR 488R (a) and ospC D1572R+AgeI (b) to determine the orientation of the *flgBp*-*aphI* antibiotic resistance cassette.

The *ospC* operator consists of a set of overlapping inverted repeats that are highly conserved in *B. burgdorferi* sensu stricto strains (Fig. 1) [65]–[67]. We hypothesized that the dIR has a specific role in responding to changes in DNA supercoiling, which is known to regulate *ospC* expression [42]. Increased negative DNA supercoiling can alter DNA structure, including extrusion of cruciforms from IRs, which can have a regulatory effect on transcription [77]–[79]. We generated site-directed mutations in the native *ospC* operator on cp26 that specifically disrupted the dIR (dIR^–^) and that changed the dIR sequence but retained complementarity (dIR^+^) (Fig. 2A; sequence changes are in bold). The orientation of the kanamycin resistance cassette, *flgBp-aphI*, downstream of the *ospC* gene was determined by PCR using the primer sets a+b (Fig. 2B). Changes in the operator sequence of the mutant strains were confirmed by DNA sequencing; about one-third of the kanamycin-resistant clones contained the site-directed mutations.

### The Role of the dIR in *ospC* Expression Induced by Relaxation of DNA Supercoiling

**Figure 3:**
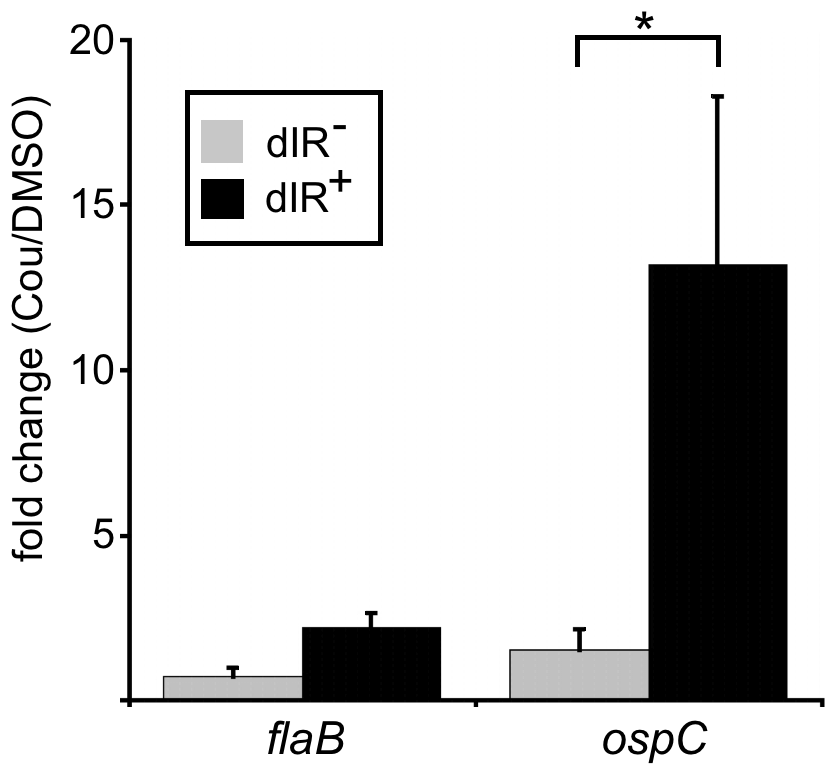
*ospC* expression induced by relaxation of supercoiling is dependent on the dIR of the operator. qRT-PCR analyses of *flaB* (gray bars) and *ospC* (black bars) mRNA levels from strains grown to late log phase at 23°C in 10 ng ml^−1^ coumermycin A_1_ (Cou) or in DMSO as a solvent control. Values represent the mean and error bars the SE of the mean from three independent experiments. * = *P*<0.05 by an unpaired *t*-test.

We have previously shown that a decrease in negative DNA supercoiling causes an increase in *ospC* transcription [42]. The antibiotic coumermycin A_1_ relaxes DNA supercoiling by inhibiting DNA gyrase [80]–[83]. Wild-type and mutant strains were grown at 23°C in the presence of coumermycin A_1_ or DMSO (solvent only control) until late log phase to examine if the increase in *ospC* expression by the relaxation of supercoiling is mediated by the dIR of the operator. Transcript levels of *ospC* and *flaB* were measured by qRT-PCR. The fold increase in *ospC* transcript in the dIR^+^ strain grown in coumermycin A_1_, compared to the solvent control, was significantly greater than that seen in the dIR^−^ strain (about thirteenfold compared to less than twofold) (Fig. 3). The change in *flaB* transcript levels was about twofold or less for both the dIR^+^ and dIR^−^ strains (Fig. 3). These data suggest that the ability to form the dIR is an important regulator of coumermycin A_1_-mediated *ospC* expression.

**Figure 4:**
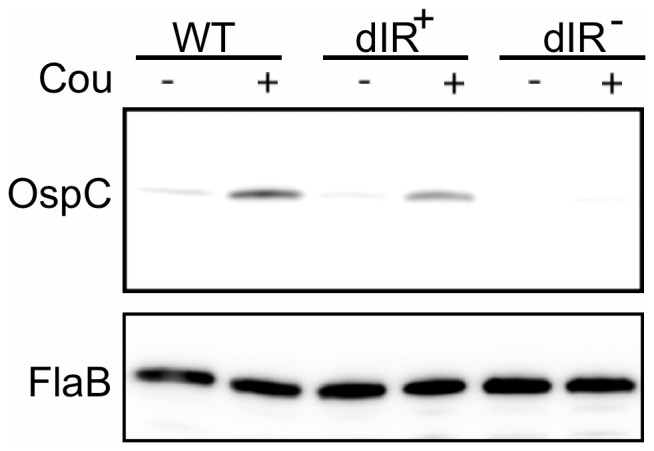
The role of the dIR in OspC synthesis mediated by relaxation of DNA supercoiling. Immunoblot analysis of whole-cell lysates from strains grown to late log phase at 23°C in 10 ng ml^−1^ coumermycin A_1_ (Cou) (+) or in DMSO as a solvent control (−). Membranes were probed with antibodies against OspC (upper panel) or FlaB (lower panel).

To examine if the changes in *ospC* transcript levels in response to coumermycin A_1_ treatment were reflected in OspC protein levels, total cell lysates were analyzed by Western blot using polyclonal antibodies to OspC to determine the levels of OspC synthesis. OspC levels increased in the wild-type 297 strain in response to relaxation of supercoiling when grown at 23°C in the presence of coumermycin A_1_ compared to cultures grown in the DMSO control (Fig. 4). Very little OspC was produced when the dIR^−^ strain was grown in coumermycin A_1_ compared with the wild-type strain and the dIR^+^ strain (Fig. 4). We have confirmed these results with a second independently constructed clone of each of the mutant strains (data not shown). Taken together, these results suggest that the ability to form the dIR is more important than the actual sequence, at least in regard to the nucleotides that we mutated. These data imply that the increase in OspC levels stimulated by relaxation of DNA supercoiling is mediated via the dIR of the operator and that the secondary structure of the operator, rather than its sequence, plays a dominant role in regulating OspC levels.

### The Role of the dIR in Temperature-regulated OspC Synthesis

**Figure 5:**
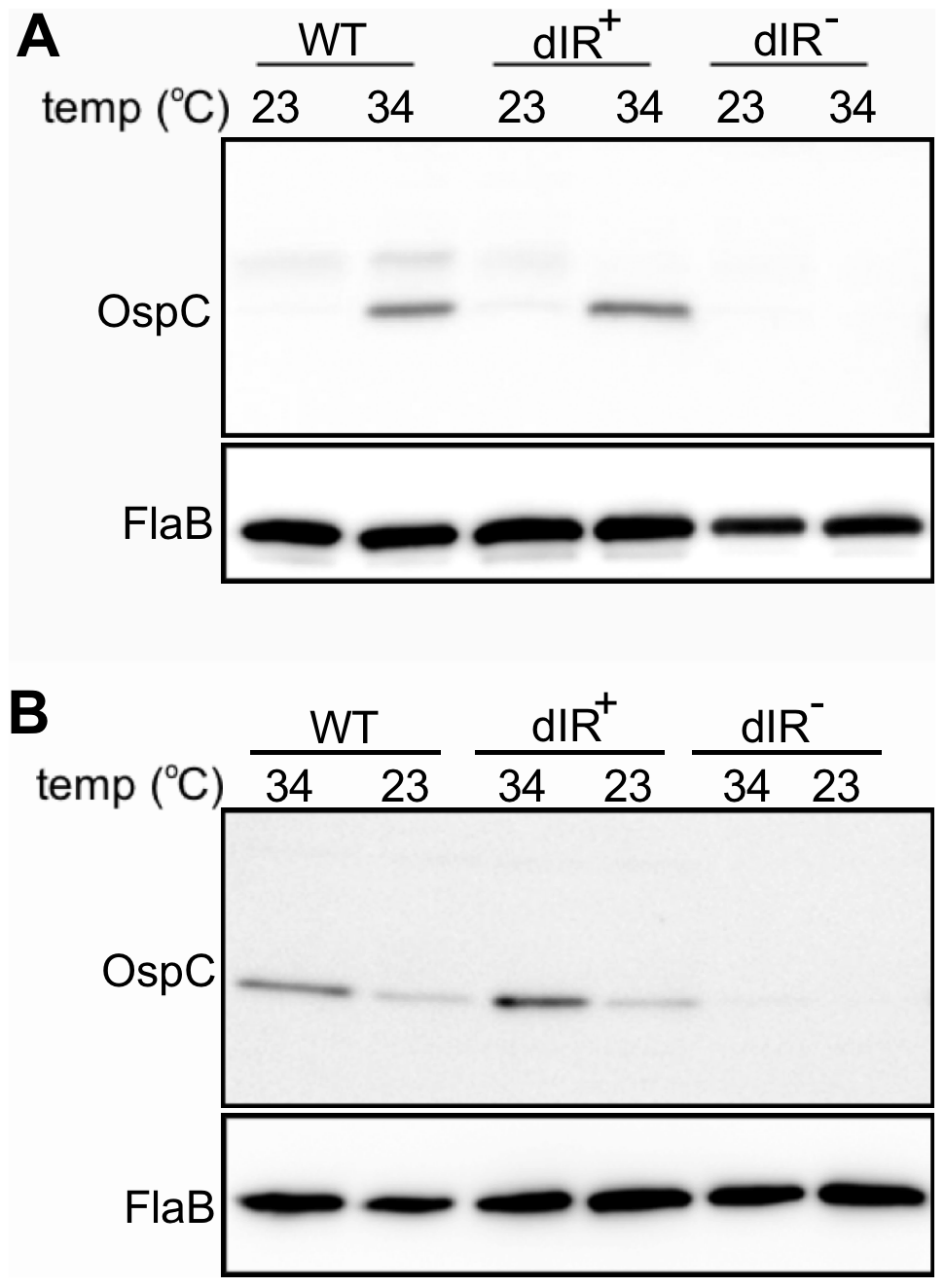
The dIR is required for OspC synthesis regulated by temperature. (A) Immunoblot analysis of whole-cell lysates from strains grown at 23°C and then temperature shifted to 34°C and grown to late log phase. The wild-type parental strain (297 WT) and the strain with a wild-type *ospC* operator linked to the antibiotic resistance cassette (WT) are controls. (B) Immunoblot analysis of whole-cell lysates from strains grown at 34°C and then temperature shifted to 23°C and grown to late log phase. Membranes were probed with antibodies against OspC (upper panels) or FlaB (lower panels).

A temperature shift from 23°C to 34°C is commonly used *in vitro* to mimic an environmental signal during tick feeding that induces virulence gene expression [8], [40]. To assess the role of the dIR in temperature-regulated OspC expression, cultures were grown at 23°C, shifted to 34°C and grown to late log phase. Total cell extracts were analyzed by Western blot. The wild-type 297 strain increased OspC synthesis upon a temperature shift (Fig. 5A). Similar to the coumermycin A_1_ treatment, OspC was not induced in response to temperature shift when the dIR was disrupted, while regenerating the complementarity of the dIR restored the ability to respond to increased temperature with increased OspC levels in the dIR^+^ strain (Fig. 5A). A second clone of each mutant, from an independent transformation, showed the same pattern of OspC levels during temperature shift (data not shown).

We next assayed if the dIR was also involved in reducing OspC levels at 23°C. Cultures were grown to late log phase at 34°C and then passaged and grown at 23°C to late log phase. Again, changing the dIR sequence but maintaining complementarity in the dIR^+^ strain allowed for the reduction in OspC levels similar to the wild-type strain shifted to 23°C (Fig. 5B).

### The Role of the dIR in pH-regulated OspC Synthesis

**Figure 6:**
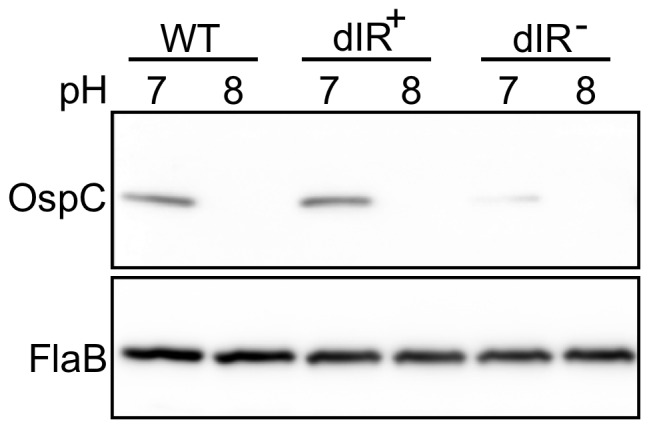
The role of the dIR in OspC synthesis mediated by pH increase. Immunoblot analysis of whole-cell lysates from strains grown to late log phase at 34°C at pH 7.0 or shifted to pH 8.0. Membranes were analyzed by probing with antibodies against OspC (upper panel) or FlaB (lower panel).

OspC levels have also been shown to be regulated by pH [43], [44], which is considered an environmental signal that changes during tick feeding: reducing the pH to 7.0 increases and raising the pH to 8.0 decreases OspC levels. To examine if pH-regulated OspC expression is mediated through the dIR, cultures were grown at 34°C in medium at pH 7.0 and then passaged into medium at pH 8.0. Total cell extracts were collected at late log phase and OspC levels were analyzed by Western blot. The dIR^–^ strain did not show increased OspC levels at pH 7.0 and the dIR^+^ strain behaved like the wild-type strain in response to changing the pH (Fig. 6). Thus, all the signals examined, DNA supercoiling, temperature, and pH, control OspC levels through the dIR of the operator *in vitro*, suggesting a common mechanism, and the complementarity of the dIR may be more important than the specific sequence.

## Discussion

Induction and repression of *ospC* transcription are crucial for *B. burgdorferi* to establish and maintain, respectively, an infection in mammals. A number of external factors, including temperature, pH, oxygen, carbon dioxide, acetate, and transition metals, regulate *ospC* expression [40]–[48], [72], [84]. Induction of *ospC* expression is generally accepted to be dependent on the RpoN-RpoS sigma factor cascade [49], which includes the regulatory proteins Rrp2 [51]–[54] and BosR [55]–[58]. Considerably less is known concerning the repression of *ospC* transcription, including the signals and accessory proteins involved. Here we show that the *cis*-acting dIR of the *ospC* operator functions to control expression and our data indicate that the base-pairing potential of the two halves of the inverted repeat, rather than the specific sequence, is essential for induction, thus providing a level of *ospC*-specific regulation downstream of RpoS.

The large intergenic region upstream of the *ospC* gene contains the operator and is highly conserved among *B. burgdorferi* sensu stricto strains, much more so than the *ospC* gene itself, likely indicating selective pressure on the regulation of *ospC* expression [65], [67]. Certain features of the operator, including the proximal IR (pIR), are broadly conserved throughout *B. burgdorferi* sensu lato genospecies, but, inexplicably, the dIR does not overlap the pIR in *B. afzelii* strains and is not even obviously present in *B. garinii* strains [67], suggesting alternate modes of gene regulation. Although we and others previously had shown that deleting the operator has little effect on the regulation of *ospC* transcription [68], [69], our current results more closely agree with Eggers et al. [64], who showed that the entire operator was required for full *ospC* expression in response to a temperature shift. These data suggest that the dIR plays a role in *ospC* regulation. This discrepancy may be explained by the differences in the experimental approaches between the studies. In the present work, we have utilized a more precise method to dissect the operator: site-directed mutations were generated in *cis* in the endogenous operator on cp26, while the other studies utilized truncated operator mutants in *trans* on a shuttle vector in an *ospC* null background. Thus, *ospC* expression in *trans* from a plasmid much smaller than cp26 (7 kb compared to 26 kb), albeit still circular, with a strong promoter fused to a selectable marker, may affect operator function, especially when DNA topology is likely involved [42], [68], [85]. In fact, OspC levels expressed in *trans* were elevated at 23°C compared to wild type, even though the plasmid-borne *ospC* contained the entire operator region [68].

Mutations that disrupt the dIR, but maintain the pIR (dIR^−^ strain) prevent an increase in OspC levels in response to temperature, pH or relaxation of supercoiling, suggesting that all these signals function through a similar mechanism. Thus, the dIR is required for an increase in the amount of OspC. The finding that the *ospC* induction by relaxation of supercoiling at 23°C with coumermycin A_1_ (Fig. 3) depends on the dIR suggests that DNA topology has a regulatory role. These data imply that the regulatory element may be the DNA structure rather than the sequence, although we may not have mutated the nucleotides in the dIR^+^ strain that are important in regulation. We propose that the inverted repeats mediate the effect of DNA supercoiling, possibly by extruding a cruciform, or bind a *trans*-acting factor that recognizes an alternative DNA secondary structure. This provides a molecular mechanism for our previous observation that relaxation of supercoiling induces *ospC* expression [42].

Finally, we are aware of only a handful of studies in which site-directed mutations were introduced in *cis* into the genome of *B. burgdorferi* [51], [73], [86], [87] and this work provides an important caveat for interpreting genetic experiments involving introduction of DNA in *trans*, albeit the methodology is considerably more convenient. Our results add another level of complexity to *ospC* regulation suggesting that the DNA structure of the operator serves to mediate the external signals affecting expression.
